# Bispecific antibody targeting shared indel-derived neoantigen of APC

**DOI:** 10.3389/fimmu.2025.1574958

**Published:** 2025-05-15

**Authors:** Clara Effenberger, Xiaojing Wu, Peng Zhao, Saki Matsumoto, Yusuke Nakamura, Kazuma Kiyotani

**Affiliations:** ^1^ Project for Immunogenomics, Cancer Precision Medicine Center, Japanese Foundation for Cancer Research, Tokyo, Japan; ^2^ Laboratory of Immunogenomics, Center for Intractable Diseases and ImmunoGenomics (CiDIG), National Institutes of Biomedical Innovation, Health and Nutrition (NIBN), Ibaraki-shi, Osaka, Japan; ^3^ Department of Human Genetics and Disease Diversity, Graduate School of Medical and Dental Sciences, Institute of Science Tokyo, Tokyo, Japan

**Keywords:** cancer immunotherapy, frameshift mutations, neoantigens, phage display library, single-chain variable fragments, single-chain diabody, TCR-like antibodies

## Abstract

T cells play a pivotal role in cancer immunotherapy by recognizing tumor-specific neoantigens presented on HLA molecules, which are specifically expressed on cancer cells. While neoantigens are generally unique to individual cancers, certain neoantigens, known as ‘shared neoantigens’ that are common in a subset of cancer patients, represent promising immunotherapeutic targets. We previously identified an immunogenic shared frameshift neoantigen, 1472SP2, derived from recurrent frameshift indel mutation cluster (APC-F2-1472*) in the *APC* gene and presented on HLA-A24:02. In this study, we attempted to identify an antibody targeting a complex formed by the APC 1472SP2 neoantigen and HLA-A24. Using the phage display library screening, we isolated single-chain variable fragments (scFvs) that specifically recognize the 1472SP2/HLA-A24 complex. We then designed a bispecific antibody (BsAb) that would connect T cells via an anti-CD3 scFv to the cancer-specific 1472SP2 presented on the HLA-A24 molecule. ELISA analysis revealed that BsAb specifically recognized both 1472SP2-HLA-A24 monomer and CD3 protein. When T cells were co-cultured with antigen-presenting cells expressing HLA-A24:02, IFN-γ release and cytotoxicity were observed only in the presence of 1472SP2-BsAb, indicating that the 1472SP2-BsAb effectively activated T cells to lyse target cells presenting this neoantigen. This approach implies an off-the-shelf, cancer selective approach to target cancers expressing shared neoantigens for patients who are difficult to treat with conventional therapies.

## Introduction

Cancer immunotherapy has transformed cancer treatment, significantly improving the prognosis of cancer patients. Immune checkpoint inhibitors (ICIs), in particular, have advanced cancer treatment by reinvigorating T-cell responses. However, the response rates to ICIs are still limited to only 10%-40% of cancer patients, highlighting the need for novel strategies to further enhance anti-tumor immune activity such as cancer vaccines or adoptive cell therapies. In these cancer immunotherapies, T cells play a crucial role by recognizing cancer-specific neoantigens, which are derived from somatic mutations in cancer cells. Neoantigens serve as specific targets for T-cell-mediated immune responses with minimizing off-target effects.

Neoantigens presented on HLA molecules, in the form of a neoantigen peptide-HLA complex, are specifically recognized by T cell receptors (TCRα and TCRβ heterodimers) expressed on the surface of T cells (1, 2). Recently, TCR-like antibodies, also known as TCR-mimic antibodies, have emerged as a novel class of agent that targets the peptide-HLA complex (3). TCR-like antibodies have advantages, including the higher affinity compared with conventional TCRs and being able to target intracellular proteins in cancer cells, whereas traditional antibody drugs can target only cell-surface molecules. TCR-like antibodies can be used as various therapeutic formats such as full-length antibodies, antibody-drug conjugates, and bispecific antibodies (BsAbs), or cell-based therapies using chimeric antigen receptors (CARs) (3–9).

In neoantigen-directed immunotherapy, BsAbs are designed to bind both a tumor-specific antigen on a cancer cell and the CD3 protein on T cells, to effectively bridge T cells to the tumor cell and to promote an immune attack on cancer cells without damaging normal cells. These BsAbs can be produced as off-the-shelf reagents and be theoretically applied for treating any patients with tumors containing the targeted antigens. For instance, the bispecific T-cell engager (BiTE) blinatumomab, which targets CD19-expressing tumors, has demonstrated the high efficacy in hematologic malignancies (10). In addition, the EGFR/c-MET immunoglobulin (IgG)-like BsAb amivantamab was approved for non-small cell lung cancer (11).

Through the screening of shared neoantigens derived from recurrent somatic mutations common among multiple cancer patients, we previously reported the identification of shared neoantigen derived from FGFR3 Y373C (12). Similarly, several shared neoantigens have been identified, but most studies including ours focused on single-nucleotide variant (SNV)-derived shared neoantigens (13–16). In our previous report (17), we demonstrated that frameshift insertions or deletions (indels) create novel open reading frames (ORFs), encoding unique protein sequences downstream of the mutation site, and that frameshift mutation clusters (FSCs) consisted of indels at multiple sites that share partly the common ORFs and premature stop codon, can sometimes generate partially-identical immunogenic peptides. Notably, we identified two FSCs such as APC-F2-1472* and APC-F3-1512*, which produced HLA-A24:02-restricted immunogenic neoantigen peptides (1472SP2 and 1512SP3) with the possible frequencies of 4.9% and 1.5%, respectively, among patients with colorectal cancers (CRCs). We also confirmed that TCR-engineered T (TCR-T) cells specific to these APC frameshift neoantigens demonstrated specific cytotoxicity against target cells, suggesting that these frameshift neoantigens could serve as effective targets. In this study, we utilized phage display library to isolate TCR-like antibodies that selectively target the HLA-A24:02-restricted APC frameshift neoantigen 1472SP2, and successfully developed an 1472SP2-BsAb capable of engaging 1472SP2/HLA-A24 complex and T cells that induced killing of target cells expressing the 1472SP2 neoantigen. This TCR-like antibody approach provides a targeted therapeutic strategy for cancers with *APC* mutations and holds a potential promise of novel treatment for a subset of colorectal cancer patients.

## Materials and methods

### Cells

Human peripheral blood mononuclear cells (PBMCs) from healthy donors were purchased from Cellular Technology Ltd. (Shaker Heights, OH, USA). CD8+ T cells were isolated from PBMCs using the Dynabeads CD8 Positive Isolation Kit (ThermoFisher Scientific, Carlsbad, CA, USA), expanded using Human T-Activator CD3/CD28 Dynabeads (ThermoFisher Scientific), and kept at -80°C until their use. The cells were cultured in AIM-V medium supplemented with 5% human AB serum and 200 IU/mL of IL-2 (R&D Systems, Minneapolis, MN, USA) at 37°C under 5% CO_2_ one day before starting co-culture experiments. The study protocol was approved by the Institutional Review Board of the Japanese Foundation for Cancer Research (2018-GA-1021) and National Institutes of Biomedical Innovation, Health and Nutrition (B2023-056). This study was conducted in accordance with the ethical principles outlined in the Declaration of Helsinki.

C1R cells (B lymphoblast cells lacking endogenous HLA-A and HLA-B expressions) were purchased from the American Type Culture Collection and were cultured in RPMI1640 supplemented with 10% FBS. C1R cells stably expressing HLA-A24:02 (C1R-A24) that were established in our previous study were used as antigen presenting cells (18). Expi293F cells were purchased from ThermoFisher Scientific and were maintained under the recommended condition.

### Peptides and peptide-HLA monomers

**Figure 1:**
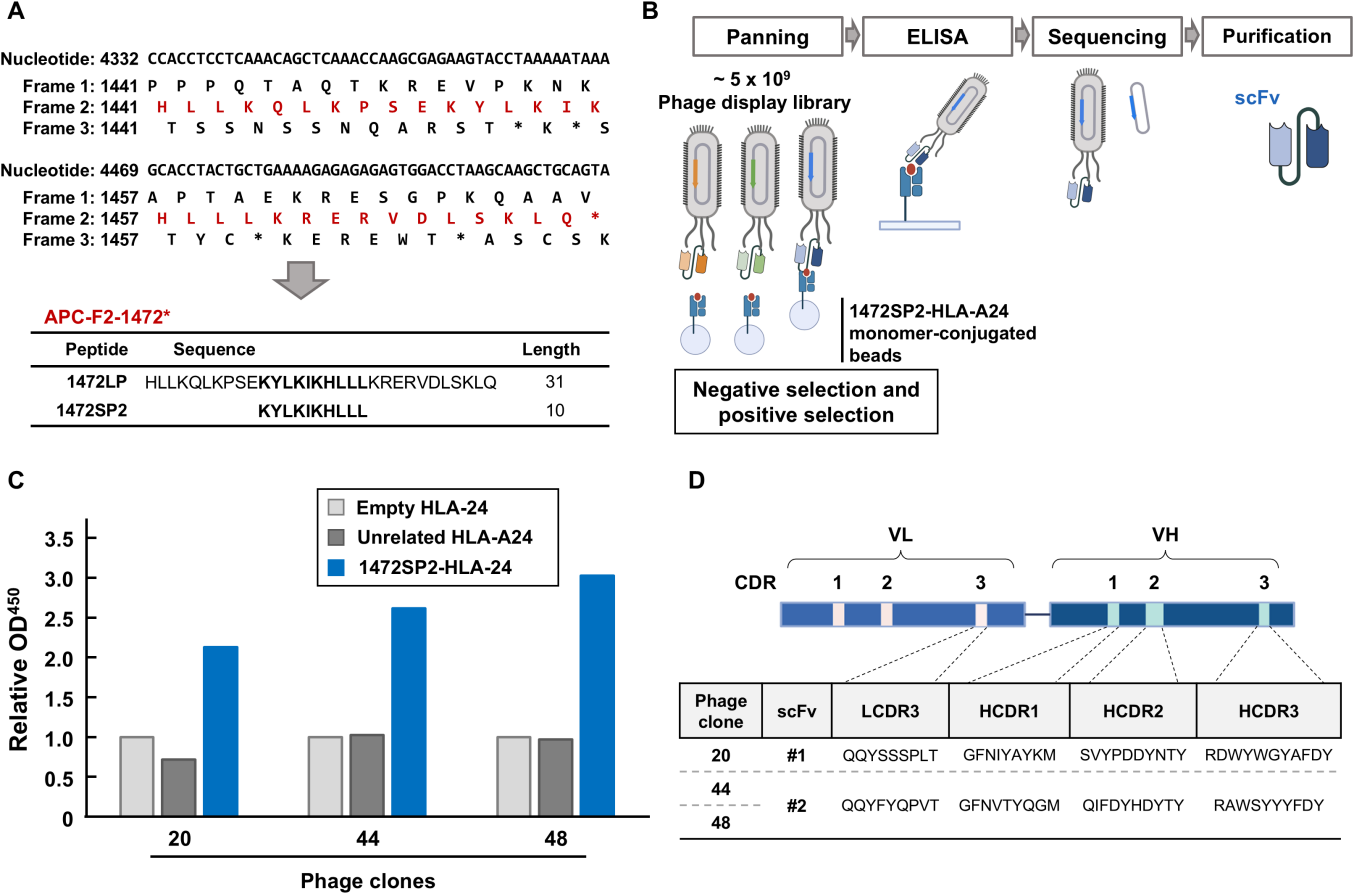
Identification of APC 1472SP2-HLA-A24 specific scFv by phage display library screening. **(A)** Nucleotide, protein and potential neoantigen peptide sequences around APC-F2-1472* clusters. Frame 1 is defined as the reference open reading frame. Frames 2 and 3 are defined as the frames with 3n+2-bp insertion or 3n+1-bp deletion, and 3n+1-bp insertion or 3n+2-bp deletion, respectively. Asterisks (*) represent a premature termination codon. **(B)** Schema to generate single-chain variable fragments (scFvs) specific to APC 1472SP2/HLA-A24 complex. Through several rounds of phage panning, using negative and positive selections, followed by ELISA analysis, phage clones displaying specific scFv to target are enriched. DNA sequences of the selected scFvs are subsequently cloned and expressed to generate scFv proteins. **(C)** Screening of specific phage clones to the 1472SP2/HLA-A24 complex by ELISA. 1472SP2-HLA-A24, unrelated 1512SP3-HLA-A24 or empty HLA-A24 monomers were bound to the plate and then incubated with supernatant of the phage clones, followed by detection with an anti-M13 antibody. Optical density at 450 nm was measured on a spectrophotometric plate reader, and was normalized to the empty HLA-A24 monomer. **(D)** scFv sequences obtained from phage clones 20, 44, 48, specific to APC 1472SP2-HLA-A24 monomer. CDR, complementarity determining region.

Short peptides of 1472SP2 (KYLKIKHLLL) and long peptide of 1472LP (HLLKQLKPSE**KYLKIKHLLL**KRERVDLSKLQ) within the APC-F2-1472* cluster and 1512SP3 (KYSRWIFLF) within the APC-F3-1512* cluster were synthesized at a purity of >95% (GenScript, Piscataway, NJ, USA; **Figure 1A**). Alanine-substituted 1472SP2 peptides were synthesized at a purity of >80% (GenScript). Biotinylated peptide-HLA monomers composed of an HLA-A24:02 molecule with the 1472SP2 or the unrelated 1512SP3 peptide that was also predicted as another APC frameshift neoantigen presented on HLA-A24, were purchased from Tetramer Shop (Kongens Lyngby, Denmark) or MBL (Tokyo, Japan). An empty HLA-A24:02 monomer was obtained from Tetramer Shop.

### Selection of phage clones

For selection of phage clones, two fUSE5 phage display libraries, which were kindly provided from Dr. Bert Vogelstein, were used (19). Phages displaying specific single-chain variable fragment (scFv) to the 1472SP2-loaded HLA-A24 monomer were selected using previously published methods (19–21). Briefly, the library was amplified in *E. coli* SS320 and was purified by polyethylene glycol/NaCl precipitation. Negative selection was performed against MyOne T1 streptavidin magnetic beads (ThermoFisher Scientific), heat-denatured HLA and unrelated peptide-HLA, followed by positive selection for the mutant peptide-HLA (1472SP2/HLA-A24) complex. Eluted phages were amplified through up to six rounds of selection, using a fraction of the phage from each previous round as input for the next. In a competition step (rounds 2-4), 1472SP2- and unrelated-1512SP3-loaded HLA-A24 monomers were co-incubated with the phage, and phage clones specific to 1472SP2-HLA-A24 were selectively retrieved. After the selection process, monoclonal phages were obtained by producing phages from bacteria transduced at limiting dilutions.

### Enzyme-linked immunosorbent assays

Binding of the phage to peptide-HLA complexes were evaluated by ELISA. In brief, 50 ng of a biotinylated peptide-HLA-A24 or an empty HLA-A24 monomer, or 25 ng of human recombinant CD3ϵ/δ (Acro Biosystems, Newark, DE, USA) were added to streptavidin-coated 96-well plates (ThermoFisher Scientific) in 50 μL of blocking buffer, consisted of PBS with 0.5% BSA, 2 mM EDTA and 0.1% sodium azide at 4°C overnight. After washing with 1× TBST (TBS with 0.05% Tween-20) six times, the bound phages were identified with anti-M13 antibody (Sino Biological, Beijing, China). Similarly, plate-bound scFvs and BsAbs were detected with anti-His antibody (Abcam, Cambridge, UK). Absorbance at 450 nm was measured with a Victor Multilabel Plate Reader (Perkin Elmer, Waltham, MA, USA).

### Recombinant scFv and BsAb production

After the phages specifically bound to 1472SP2-HLA-A24 monomer were selected, the phage DNA was PCR-amplified by the primers flanking the CDRs and then sequenced. The obtained scFv sequences were cloned into pET-44a(+) vector (Novagen, Milwaukee, WI, USA). Recombinant scFv was produced in *Escherichia coli* with periplasmic expression, followed by the purification using HisPur Ni-NTA Resin (ThermoFisher Scientific). The BsAb constructs were obtained by inserting the assembled synthetic gene 1472SP2-CD3(UCHT1)-single chain diabody (scDb) and C-terminal 6×His tag into pcDNA3.1 mammalian expression vector (ThermoFisher Scientific). In this study, we used scFv sequence of the clone UCHT1 as CD3 antibody reported in previous studies (20, 21), since UCHT1 showed the strongest affinity to CD3ϵ/δ. Recombinant BsAbs were produced by secretion from Expi293F and then purified using HisPur Ni-NTA Resin (ThermoFisher Scientific). Proteins were quantified with the Pierce BCA Protein Assay Kit (ThermoFisher Scientific). SDS-PAGE and western blotting analysis were performed to identify the components of the purified product with a common procedure. Precision Plus Protein WesternC standards (BioRad, Hercules, CA, USA) were used as a size marker.

### Enzyme linked immuno-spot and cytotoxicity assays

To evaluate the effects of BsAbs on cytokine release and the cytotoxic activity induced by CD8+ T cells in response against target cells, co-culture experiments were conducted. Briefly, C1R-A24 cells used as target cells were pulsed with 1472SP2 or unrelated 1512SP3 at 37°C for 16 h. CD8+ T cells, isolated and expanded from healthy donors’ PBMCs, were seeded at a density of 1 × 10^5^ cells/well and co-cultured with the target cells at a 2:1 ratio (CD8+ T cells: target cells, respectively) in the presence of the BsAbs or an isotype control at the indicated concentrations. Co-culture was conducted in the presence of 200 IU/mL IL-2 at 37°C for 24 h in 96-well plate. Interferon (IFN)-γ secretion from T cells were detected by ELISPOT assay using Human IFN-γ ELISpotPRO kit (MABTECH, Stockholm, Sweden) according to the manufacturer’s instruction. Spots were captured and analyzed by an automated ELISPOT reader, ImmunoSPOT S4 (Cellular Technology Ltd, Shaker Heights, OH, USA) and the ImmunoSpot Professional Software package, Version 5.1 (Cellular Technology Ltd).

Cell viability was assayed by CellTiter-Glo Luminescent Cell Viability Assay (Promega, Madison, WI, USA) according to the manufacturer’s instructions. Cytotoxicity was calculated by taking the luciferase signal of a given well, subtracting the luciferase signal of the CD8+ T cell-only wells, and normalizing to the luciferase signal of the wells without BsAbs.

### Statistical analysis

Statistical analyses were performed with Prism 8 (GraphPad software). Data are presented as means ± SD unless otherwise specified. Student’s *t*-test was performed to compare the data in ELISA, ELISPOT and cytotoxicity assays. A *P* value of <0.05 was considered as statistically significant.

## Results

### Identification of scFv-expressing phage clones specific to APC frameshift neoantigen 1472SP2

In our previous study (17), we identified two potential genetic regions termed (frameshift mutation clusters) FSCs in the *APC* gene, where frameshift mutations generating new peptide sequences common in multiple cancers are accumulated. In these FSCs, we identified two immunogenic peptides, 1472SP2 and 1512SP3, presented on HLA-A24:02 molecules found at 4.9% and 1.5% of CRCs, respectively, in TCGA database. In this study, we attempted to isolate scFvs that can specifically bind to 1472SP2 frameshift neoantigen peptide on the HLA-A24 molecule through phage display library screening (**Figure 1B**), using two scFv-phage display libraries, both based on the humanized 4D5 framework with introduction of variability in four CDR3s (L3, H1, H2 and H3) or in five CDR3s (L2, L3, H1, H2 and H3), respectively, using trinucleotide mutagenesis technology (19, 22, 23). We conducted over six rounds of panning, using three distinct amplified phases: an enrichment phase (round 1), a competitive phase (rounds 2-4), and a final selection phase (rounds 5 and 6), which enabled us to gradually enrich mutant peptide-HLA-A24-specific phages.

After the six rounds of panning, we picked up a total of 63 phage clones and evaluated their binding specificity against the 1472SP2-HLA-A24 monomer by ELISA (**Supplementary Figure 1**). Among the 63 clones we examined, we found three phage clones, clones 20, 44 and 48, showed substantially greater binding to only 1472SP2-HLA-A24, but not to the empty HLA-A24 or the unrelated 1512SP3-HLA-A24 monomers (**Figure 1C**). We then performed DNA sequencing of these three clones and identified two different scFv sequences (scFv #1 from clone 20 and scFv #2 from clones 44 and 48; **Figure 1D**).

### Evaluation of specific binding of scFvs to APC 1472SP2-HLA-A24 monomer

**Figure 2:**
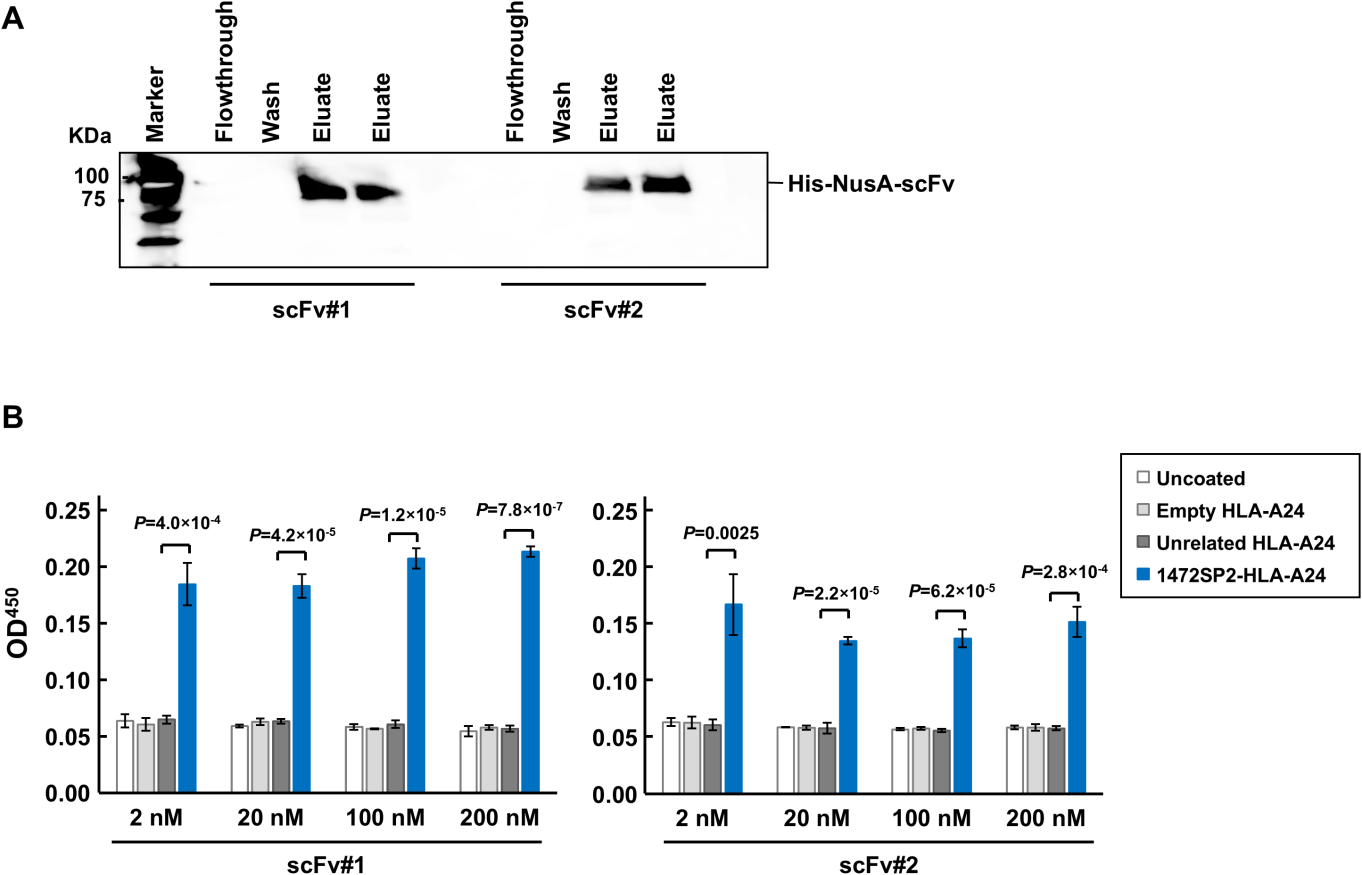
Characterization of APC 1472SP2 scFv by ELISA. **(A)** Western blot analysis of the purified single-chain variable fragments (scFvs) from the clonal samples, detected using an anti-His antibody. **(B)** Selective binding of the purified APC 1472SP2 scFvs to 1472SP2/HLA-A24 complex by ELISA. 1472SP2-HLA-A24, unrelated 1512SP3-HLA-A24 or empty HLA-A24 monomers were bound to a streptavidin plate and then incubated with 1472SP2 scFvs at different concentrations (2 nM, 20 nM, 100 nM and 200 nM), followed by detection with an anti-His antibody. Optical density at 450 nm was measured on a spectrophotometric plate reader. Data are represented as mean ± SD (*n* = 3).

To generate scFvs uncoupled from the M13 pIII protein, we purified ssDNA from the phage, amplified the scFv region by PCR, and then cloned into the pET-44 bacterial expression vector. Using Western blot analysis of the purified scFvs, we confirmed their expression by detecting the protein band at approximately 80 kDa, corresponding to the scFvs (**Figure 2A**). ELISA showed that both scFvs exhibited a binding response to the 1472SP2-HLA-A24, with no binding to unrelated 1512SP3-HLA-A24 monomer at concentrations up to 200 nM (**Figure 2B**). We selected scFv #1, which revealed a stronger signal at lower concentrations, for further evaluation.

### Evaluation and characterization of BsAb binding to APC 1472SP2-HLA-A24 monomer

**Figure 3:**
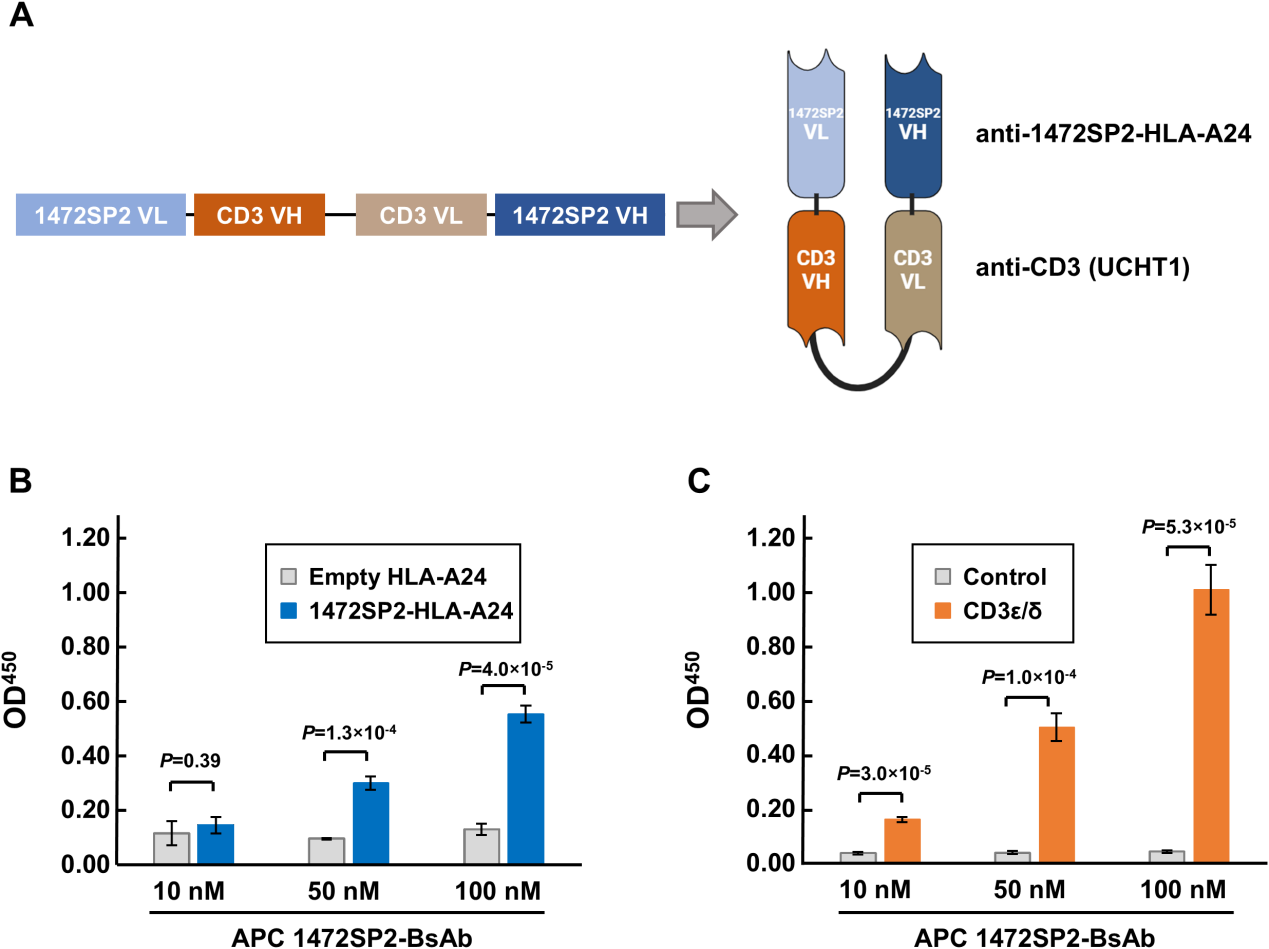
Characterization of APC 1472SP2-BsAb by ELISA. **(A)** Schematic structure of the APC 1472SP2-bispecific antibody (BsAb), consisting of the anti-1472SP2-HLA-A24 variable light chain (VL), GGGGS short linker, anti-CD3 (clone UCTH1) variable heavy chain (VH), (GGGGS)_3_ long linker, anti-CD3 VL, GGGGS short linker and anti-1472SP2-HLA-A24 VH. **(B, C)** Selective binding of the purified 1472SP2-BsAb to 1472SP2/HLA-A24 complex **(B)** and CD3 **(C)** by ELISA. Biotinylated 1472SP2-HLA-A24 or recombinant CD3ϵ/δ was coated on a streptavidin plate and then incubated with APC 1472SP2-BsAb at the different concentrations (10 nM, 50 nM and 100 nM), followed by detection with an anti-His antibody. Data are represented as mean ± SD (*n* = 3).

Various T cell-engaging BsAb formats have been developed to direct T cells to specific targets (24). In this study, we opted to use the scDb format due to its proven success in previously published works (20, 21). The anti-1472SP2 scDb consists of two distinct scFv fragments, one targeting the 1472SP2/HLA-A24 complex and the other recruiting and activating T cells by binding to the CD3 protein. We used short Gly-Ser-rich linkers to connect the variable heavy chain (VH) and variable light chain (VL) domains of the scFvs, enabling their homodimerization (**Figure 3A**). We produced anti-1472SP2-BsAb by cloning the fragments into an eucaryotic expression vector containing 6×His tag, which enabled high-level of expression and easy purification.

We first evaluated the ability of the 1472SP2-BsAb to bind to 1472SP2-HLA-A24 monomer on streptavidin-plate by ELISA, and found that the 1472SP2-BsAb interacted specifically with 1472SP2-HLA-A24 in a dose-dependent manner at concentrations up to 100 nM, but not with an empty HLA-A24 monomer (**Figure 3B**). Additionally, we confirmed that 1472SP2-BsAb, which includes anti-CD3 (clone UCHT1), dose-dependently binds to the CD3ϵ/δ complex, while no significant binding was observed to the control (**Figure 3C**), indicating proper protein folding and functionality of the APC 1472SP2-BsAb.

### Characterization of immunological function of APC 1472SP2-BsAb

**Figure 4:**
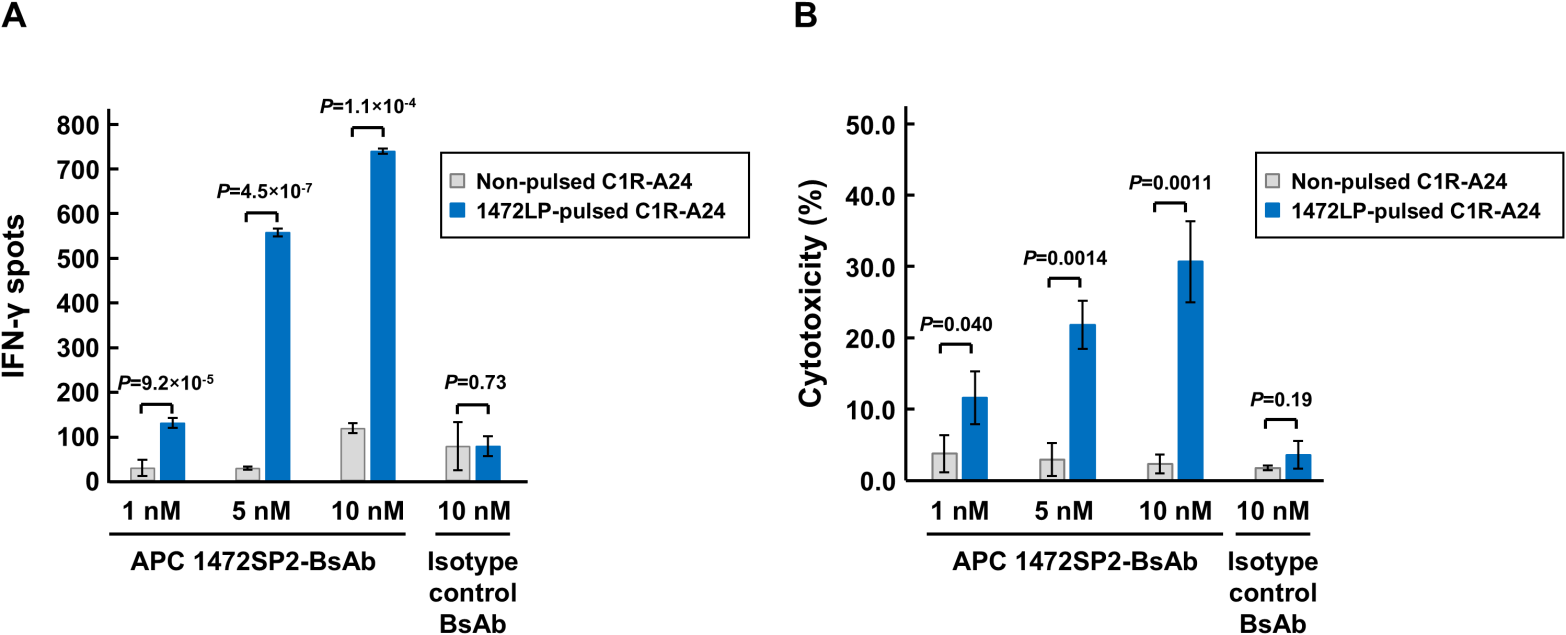
Immunological characterization of APC 1472SP2-BsAb function. **(A)** T cell activation by APC 1472SP2-bispecific antibody (BsAb) evaluated by ELISPOT assay. C1R-A24 cells were pulsed with or without 1472LP and then co-cultured with CD8+ T cells in the presence of different concentrations of 1472SP2-BsAb or an isotype control (BsAb targeting unrelated peptide-HLA-A24), and released IFN-γ was measured by ELISPOT assay. Data are represented mean ± SD (*n* = 3). **(B)** Effect of APC 1472SP2-BsAb on cell killing function of CD8+ T cells. 1472SP2-pulsed C1R-A24 cells were co-incubated with CD8+ T cells at a ratio of 2.5:1 in the presence of increasing amounts of 1472SP2-BsAb or an isotype control (BsAb targeting unrelated peptide-HLA-A24). Specific cell lysis was evaluated by means of luminescent cytotoxicity assay. Data are represented as mean ± SD (*n* = 3).

To evaluate whether the 1472SP2-BsAb could specifically recognize the mutant 1472SP2 peptide/HLA-A24 complex on the cell surface, we evaluated T cell activation by measuring IFN-γ production after co-culturing HLA-A24-expressing C1R-A24 cells, either pulsed with the 1472LP long peptide to confirm intracellular processing of the expected neoantigen, or left non-pulsed, with CD8+ T cells isolated from a healthy donor in the presence of the 1472SP2-BsAb or an isotype antibody control (**Figure 4A**). The 1472SP2-BsAb selectively induced secretion of IFN-γ only with peptide-pulsed C1R-A24 cells in a dose-dependent manner, while the isotype control BsAb did not even at a higher concentration of 10 nM, demonstrating that the 1472SP2-BsAb effectively recognized 1472SP2/HLA-A24 complex generated through endogenous antigen processing.

To demonstrate the ability of the BsAb to mediate targeted cell killing, we conducted a cytotoxicity assay after co-culture of 1472LP-pulsed or non-pulsed C1R-A24 cells with CD8+ T cells in the presence of the 1472SP2-BsAb or an isotype control at increasing concentrations (**Figure 4B**). Similarly to the ELISPOT results, we observed specific cell lysis only when we added the 1472SP2-BsAb even at low concentration of 1 nM, although we did not observe when we used the isotype control antibody.

### Potential cross-reactivity to other putative HLA-A24-binding peptides

**Figure 5:**
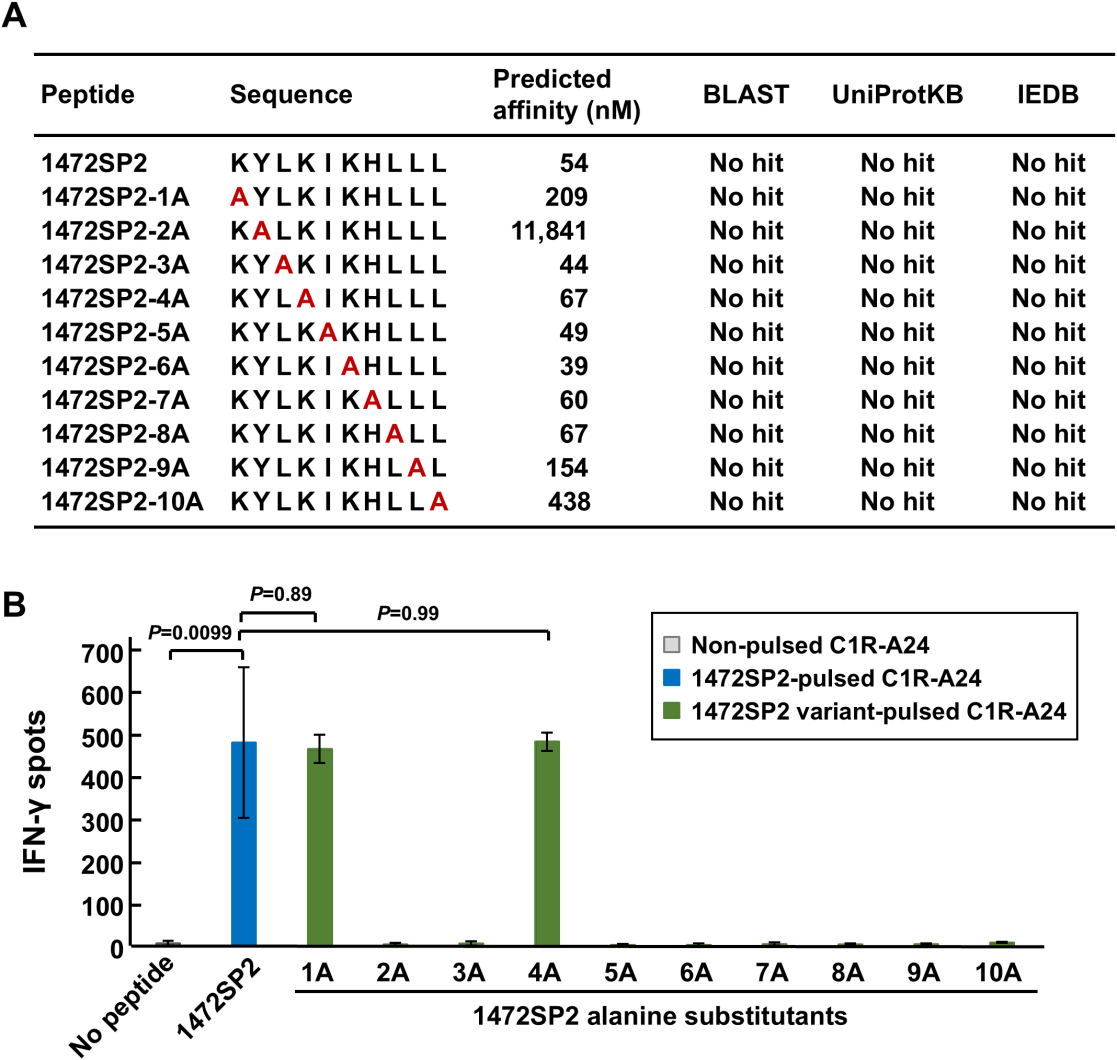
Putative cross-reactive peptides for APC 1472SP2-BsAbs. **(A)** APC 1472SP2 variant peptides with alanine substitution at a different position. Binding affinity of the variant peptides was predicted by netMHCv4.0, and searches were conducted through BLAST, UniProtKB, and IEDB to evaluate relevant hits. **(B)** Cross-reactivity of 1472SP2 variant peptides. C1R-A24 cells were pulsed with or without 1472SP2 or the alanine-substituted peptides and then co-cultured with CD8+ T cells in the presence of 5 nM of APC 1472SP2-BsAb, and IFN-γ release was measured by ELISPOT assay. Data are represented mean ± SD (*n* = 3).

To assess potential cross-reactivity, we performed the alanine scan assay by replacing each amino acid of the 1472SP2 peptide with an alanine to create a series of variant peptides (1472SP2-1A to -10A; **Figure 5A**). Since those peptides except for 1472SP2-2A were predicted to bind to HLA-A24:02 using NetMHC v4.0 (predicted IC_50_ < 500 nM), we tested immune reactivity of 1472SP2-BsAb to these variant peptides by ELISPOT assay using C1R-A24 cells. We observed that the 1472SP2-BsAb recognized two peptides, 1472SP2-1A and -4A, which substituted at position 1 or 4 to an alanine, with the IFN-γ release as similar to the original 1472SP2 peptide (**Figure 5B**). To further investigate the possibility that these two peptides are naturally presented, we searched for their sequences in the BLAST, UniProtKB human protein databases and the Immune Epitope Database (IEDB) HLA-peptide database. However, despite conducting both an exact match search and a 90% BLAST search, neither of these two peptides was found to be present in our genome, suggesting the low possibility of cross-reactivity to any proteins in human.

## Discussion

Cancer immunotherapy targeting shared neoantigens, in addition to personalized neoantigen-targeted therapies, is a promising approach because of their high cancer-specificity and a potential of a little broader therapeutic applicability. Furthermore, using this kind of approach, we are able to construct off-the-shelf types of antibody drugs, as they streamline treatment accessibility, reduce production costs, and allow rapid distribution to a subset of patient populations with the same neoantigen and the same HLA types. Therefore, in this study, we focused on the APC neoantigen, 1472SP2, generated from frameshift mutations in the *APC* gene that was frequently found in CRCs. Using phage display screening, we successfully identified a TCR-like antibody that specifically recognizes the 1472SP2/HLA-A24 complex at a concentration of 2 nM (**Figures 1**, **2**). Additionally, this discovery facilitates the development of BsAbs that recruit T cells to eliminate target cells expressing this 1472SP2 neoantigen on HLA-A24. The developed 1472SP2-BsAb demonstrated high specificity to the 1472SP2/HLA-A24 complex, activating T cells at low concentrations, and selectively targeting and attacking cells expressing 1472SP2/HLA-A24 complex on their surface in the *in vitro* experiments (**Figures 3**, **4**). To evaluate therapeutic potential of this 1472SP2-BsAb, *in vivo* studies using xenograft mouse models should be essential. For this purpose, identification of suitable cell lines expressing the relevant APC frameshift neoantigen and HLA-A24 is essential. In general, eliciting effective immune responses remains challenging in solid tumors, but it is reported that BsAbs targeting neoantigens from *TP53* and *RAS* genes have resulted in significant regression of well-established tumors in *in vivo* studies using human xenograft models (20, 21). In these studies, they compared different anti-CD3 clones, including UCHT1, OKT3 and L2K-07, and found that UCHT1-based BsAb showed a stronger immune activation. However, there is no universal “best format” for BsAbs, and the optimal anti-CD3 clone may depend on the target epitope, as OKT3-based BsAbs have been used to target antigens like B7-H3 and GD2. Although we did not conduct a systematic comparison among different anti-CD3 scFvs in combination with our APC 1472SP2-targeting arm, future optimization and head-to-head testing will be needed to refine therapeutic efficacy. Nonetheless, this approach offers a promising off-the-shelf therapeutic strategy to any HLA types by harnessing T cell-mediated cytotoxicity against cancer cells presenting shared APC and other neoantigens.

The *APC* gene, which we focused on in this study, is mutated in approximately 70-80% of CRCs (25, 26), with the majority being loss-of-function indels types concentrated in the mutation cluster region in the last exon (27). The APC 1472SP2 frameshift neoantigen we examined in this study were observed at a frequency of 4.9% in CRC patients and presented on HLA-A24, one of the most common *HLA* alleles in the Japanese population with nearly 61% frequency, while its frequency was reported to be 5-20% in Caucasians (28). Hence, this treatment could potentially be applicable to about 3% of Japanese CRC patients. Given that CRC is an increasingly prevalent cancer in Japan and worldwide, with an estimated annual incidence of approximately 2 million cases in 2022 (29). In Japan, CRC has been the most common cause of cancer death in women.

We identified TCR and TCR-like antibody for APC neoantigen 1472SP2 presented on HLA-A24:02 in the current study and in our recent paper (17). The binding between TCR and peptide-HLA complex is more specific but has lower affinity, as TCRs are naturally evolved to ensure precise immune responses while avoiding unintended interactions (30). In contrast, TCR-like antibodies are engineered for higher affinity to the peptide/HLA complex, although they may exhibit lower specificity than TCRs due to broader binding capacity. Therefore, we investigated the risk of cross-reactivity to similar peptides presented on HLA-A24 molecules (**Figure 5**), but our APC 1472SP2-BsAb showed limited cross-reactivity. Although two alanine-substituted peptides at positions 1 and 4 presented on HLA-A24 were able to be recognized by the 1472SP2-BsAb, BLAST analysis revealed no possibility of cross-reactive peptides encoded in the human genome, supporting a low risk of unintended interactions. Several reports indicated that CAR-T cells using TCR-like antibodies targeting shared antigens such as NY-ESO-1 or MART-1 exhibited effective and specific cell killing compared to TCR-T cells (31, 32). This suggests that TCR-like CAR-T cell therapy using anti-1472SP2 scFvs may also be beneficial to various types of cancer expressing 1472SP2 neoantigens and HLA-A24 by providing high specificity and minimizing the off-target effects. However, *in vivo* validation studies will be critically essential to fully assess both safety and therapeutic efficacy in a physiologically relevant setting.

In conclusion, we identified an scFv of TCR-like antibody specific to APC frameshift neoantigen 1472SP2/HLA-A24 complex, and demonstrated that a BsAb using this scFv and anti-CD3 induces immune responses and cytotoxic activity specifically to the 1472SP2 neoantigen presented on HLA-A24. These findings suggest that harnessing shared neoantigens, like APC 1472SP2, holds a promising therapeutic potential for off-the-shelf immunotherapies, offering a novel strategy for targeting common mutations across different types of cancer patients.

## Data Availability

The original contributions presented in the study are included in the article/**Supplementary Material**. Further inquiries can be directed to the corresponding author.
