# Supplementary material for: Bispecific antibody targeting shared indel-derived neoantigen of APC
Source: Front Immunol. 2025 May 15;16:1574958. doi: 10.3389/fimmu.2025.1574958 (PMC12119644; doi:10.3389/fimmu.2025.1574958)
Supplement: Supplementary file 1 [file DataSheet1.pdf]

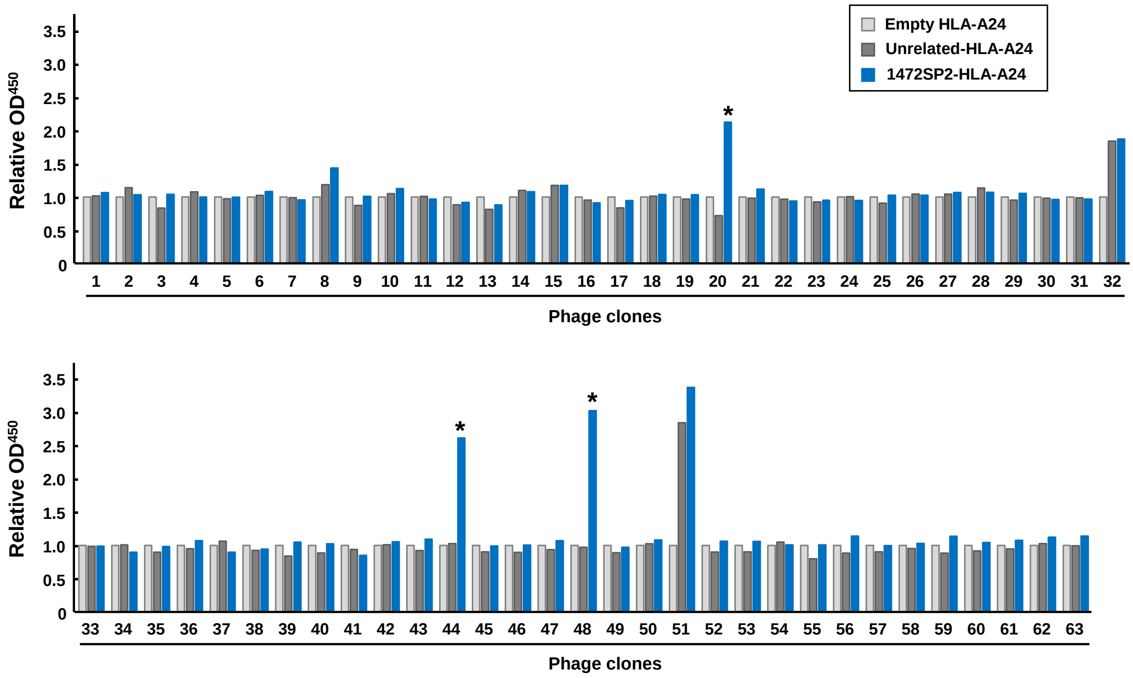

Supplementary Figure 1. Screening of phages expressing scFvs specific to APC 1472SP2/HLA-A24 complex.

1472SP2-HLA-A24, unrelated 1512SP3-HLA-A24 or empty HLA-A24 monomers were bound to the plate and then incubated with supernatant of the phage clones, followed by detection with an anti-M13 antibody. Optical density at 450 nm was measured on a spectrophotometric plate reader. Asterisks (*) represent positive clones, related to **Figure 1**.
